# High immune cell infiltration predicts improved survival in cholangiocarcinoma

**DOI:** 10.3389/fonc.2024.1333926

**Published:** 2024-05-01

**Authors:** Erkki-Ville Wirta, Säde Szeto, Hanna Koppatz, Arno Nordin, Heikki Mäkisalo, Johanna Arola, Jukka Sirén, Maarit Ahtiainen, Jan Böhm, Jukka-Pekka Mecklin, Ville Sallinen, Toni T. Seppälä

**Affiliations:** ^1^ Department of Gastroenterology and Alimentary Tract Surgery, Tampere University Hospital, Tampere, Finland; ^2^ Faculty of Medicine and Health Technology, Tampere University and Tays Cancer Centre, Tampere University Hospital, Tampere, Finland; ^3^ Applied Tumor Genomics Research Program, Research Program Unit, Faculty of Medicine, University of Helsinki, Helsinki, Finland; ^4^ Department of Abdominal Surgery, Helsinki University Hospital and University of Helsinki, Helsinki, Finland; ^5^ Transplantation and Liver Surgery, Helsinki University Hospital and University of Helsinki, Helsinki, Finland; ^6^ Department of Pathology, Helsinki University Hospital and University of Helsinki, Helsinki, Finland; ^7^ Department of Molecular Pathology, Central Finland Hospital Nova, Well Being Services County of Central Finland, Jyväskylä, Finland; ^8^ Department of Education and Science, Central Finland Hospital Nova, Well Being Services County of Central Finland, Jyväskylä, Finland; ^9^ Faculty of Sports and Health Sciences, University of Jyväskylä, Jyväskylä, Finland

**Keywords:** cholangiocarcinoma, tumor-infiltrating T-lymphocytes, immune cell score, PD-1, PD-L1

## Abstract

**Background:**

Antitumoral immune response has a crucial role in constraining cancer. However, previous studies on cholangiocarcinoma (CCA), a rare and aggressive cancer, have reported contradictory findings on the prognostic impact of tumor-infiltrating T-lymphocytes. We aimed to clarify the effect of tumor-infiltrating CD3+ and CD8+ lymphocytes and PD-1/PD-L1 expression on CCA prognosis.

**Methods:**

CD3+, CD8+, and PD-1+ lymphocyte densities, as well as PD-L1 expression rate were analyzed from stained tissue microarray samples from the tumor center and invasive margin of 47 cholangiocarcinomas. The association of CD3+ and CD8+ based Immune cell score (ICS) and its components with overall survival was evaluated, adjusting for age, sex, TNM stage, radicality of surgery, tumor location, and PD-L1 expression on immune cells.

**Results:**

Low ICS was a strong independent prognostic factor for worse overall survival (Hazard ratio 9.27, 95% confidence interval 2.72-31.64, P<0.001). Among the ICS components, high CD8+ lymphocyte infiltration at the tumor center had the most evident impact on patient outcome. PD-1 and PD-L1 expression on immune cells did not have a significant impact on overall survival alone; however, PD-L1 positivity seemed to impair survival for ICS^low^ subgroup.

**Conclusion:**

Identifying patient subgroups that could benefit from immunotherapy with PD-1/PD-L1 pathway blockade may help improve treatment strategies for this aggressive cancer. Our findings highlight the importance of evaluating the immune contexture in cholangiocarcinoma, as ICS serves as a strong independent prognostic and selective factor for patients who might benefit from immunotherapy.

## Introduction

Cholangiocarcinoma (CCA) is a rare group of malignancies originating from the epithelial cells of the biliary tree, accounting for about 3% of all gastrointestinal cancers. CCA is characterized by features of cholangiocyte differentiation and are traditionally divided in to intrahepatic (10-20% of all CCA), perihilar (50-60%), or distal disease (20-30%) according to anatomic location (1). Perihilar and distal diseases are typically mucin-producing adenocarcinomas with periductal-infiltrating or, less frequently, intraductal growth patterns. Intrahepatic tumors are more heterogeneous and can be classified according to the size of the bile duct in question. Cancers of small bile ducts invade liver parenchyma and are often mass-forming small-sized tubular or acinar adenocarcinomas with infrequent mucus secretion, while cancer originating from larger ducts resembles perihilar or distal CCA (2). Although several known risk factors for CCA exist, all causing chronic inflammation and cholestasis, most cases of CCA are considered sporadic (3).

Due to the aggressive nature of the disease, late occurring symptoms, and difficult anatomic location complicating diagnostics, only about one-third of the patients have early-stage disease still feasible for curative surgery (1). However, the recurrence rate is very high even after R0 resection (4). Five-year survival in operated perihilar or distal cancer ranges between 20-40%, and in operated intrahepatic cancer, it ranges from 10-49% (5). For locally advanced or metastatic cancer, median overall survival is less than one year with standard chemotherapy (6). Risk factors for poor survival in intrahepatic CCA include multiple tumors, regional nodal involvement, vascular invasion, large tumor size (over 5 cm), poor tumor differentiation, and R1 resection. For perihilar disease, the most important prognostic determinants are radicality of surgery, lymph node status, and tumor differentiation, and to a lesser extent, vascular or perineural invasion (4). In addition to the aforementioned factors, pancreatic involvement and T stage ≥ 3 were also prognostic for worse disease outcome in distal CCA (7).

Tumor-infiltrating lymphocytes play a crucial role in the host’s anti-tumoral response and are associated with improved prognosis in several cancers (8). However, the role of tumor-infiltrating lymphocytes in CCA is still insufficiently understood (9). To avoid immune surveillance, cancer cells exploit checkpoint inhibition pathways, such as the PD1/PD-L1 (programmed cell death protein-1/programmed cell death ligand-1) pathway, which normally regulate the magnitude of immune reactions. Immunotherapy through blockade of checkpoint molecules such as PD-1 can restore T-cell mediated anti-tumor response (10). As in many other cancers, immunotherapy has become a subject of extensive research in CCA (11), and promising results have recently been reported with PD-L1 blockade combined with standard chemotherapy gemcitabine and cisplatin (12). In this study, we aimed to determine the prognostic effect of CD3+ and CD8+ lymphocytes in surgically treated CCA from a single-center patient cohort. We also evaluated the association of PD-1+ immune cells and PD-L1 expression at the tumor site with tumor-infiltrating lymphocytes and survival in CCA.

## Materials and methods

Study population consists of cholangiocarcinomas operated at Helsinki University Hospital during 1990-2013 with adequate tumors samples available. The tissue specimens were retrieved through Helsinki Biobank. The clinical data were collected from hospital patient records. Dates of death for all patients were obtained from the Population Register Center through the electronic medical record system. Histopathological diagnoses were re-evaluated by an experienced liver pathologist (JA).

### Tissue microarray

After identifying the most suitable formalin-fixed paraffin-embedded (FFPE) tissue blocks per case, fresh slides were sectioned, stained with H&E, and digitized with a Panoramic scanner (3DHISTECH, Budapest, Hungary). Annotations for the prepared TMA were marked on the digitized slides in the CaseViewer software (3DHISTECH) in accordance with the following principles: two cores from the middle of the tumor, two cores from the tumor border (invasive margin) and two cores from the non-tumor area. The blocks were cut into 3.5μm-thick sections. Tissue microarray (TMA) blocks were prepared with a TMA Master II tissue microarrayer (3DHISTECH), containing 0.6 mm-diameter cores.

### Immunohistochemical analyses

**Figure 1:**
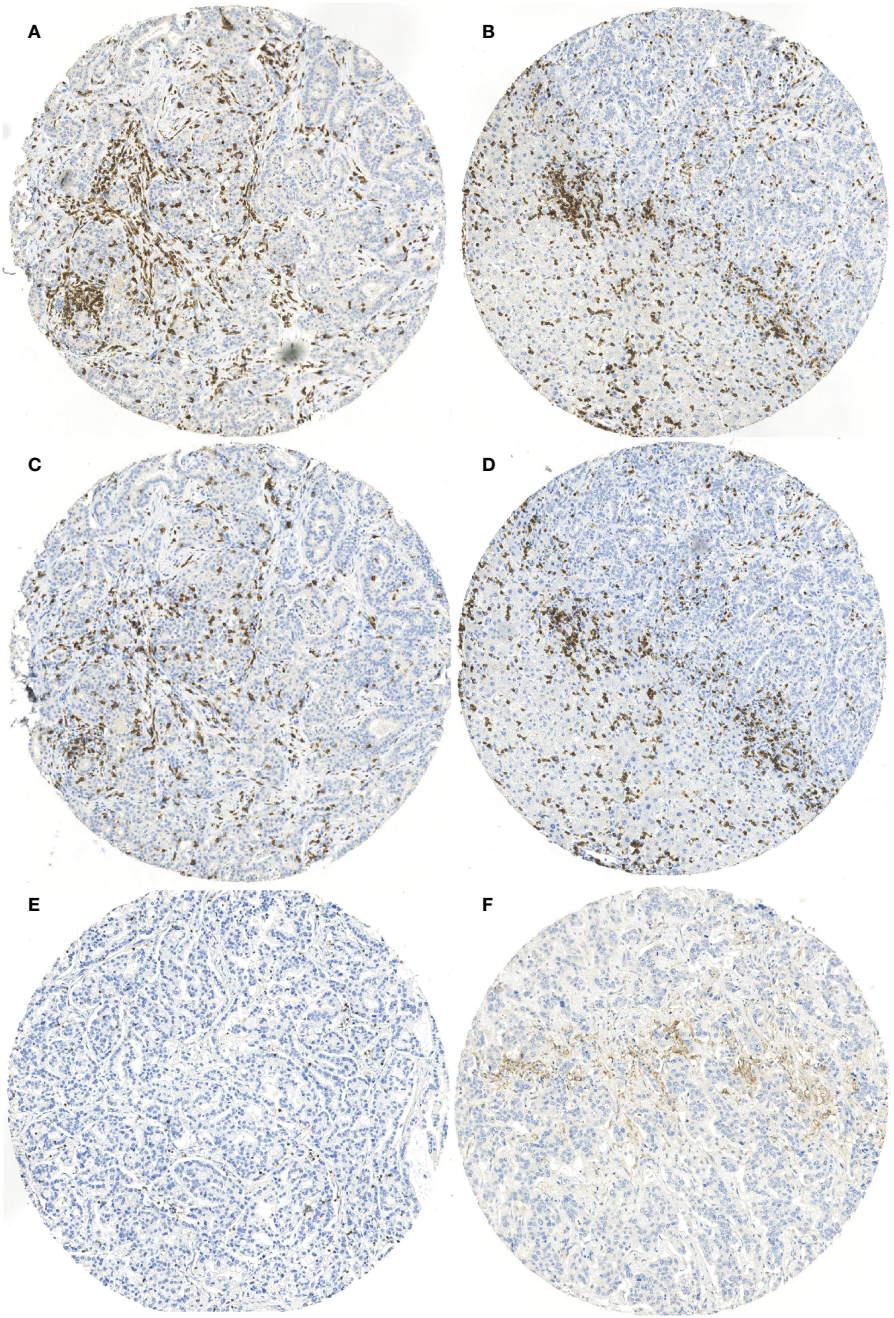
Examples of **(A)** CD3 staining from tumor center, **(B)** CD3 staining from tumor invasive margin, **(C)** CD8 staining from tumor center, **(D)** CD8 staining from tumor invasive margin, **(E)** PD-1 staining from tumor center, and **(F)** PD-L1 staining of immune cells from tumor center.

Staining for PD-1, PD-L1, and MLH1 was conducted with PD-1 (SP269, 1:50; Spring Bioscience), PD-L1 (E1L3N, 1:100; Cell Signaling Technology), and MLH1 (NCL-L-MLH1, clone ES05, 1:50; Novocastra) antibodies, using a BOND-III stainer (Leica Biosystems) as presented by Ahtiainen et al. (12) Staining for CD3 and CD8 was conducted with CD3 (LN 10, 1:50; Novocastra) and CD8 (SP16, 1:100; Thermo Scientific) antibodies, using a Lab Vision Autostainer 480 (ImmunoVision Technologies Inc.). Signal visualization was achieved using diaminobenzidine, and sections were counterstained with hematoxylin. Slides were scanned with a NanoZoomer-XR (Hamamatsu Photonics) at ×20 magnification (13). Examples of CD3, CD8, PD-1, and PD-L1 staining are shown in **Figure 1**.

Positively stained CD8 and CD3 lymphocytes and PD-1 immune cells were enumerated using QuPath, an open-source software for bioimage analysis (14). Analysis for CD3 and CD8 was conducted by two researchers (EW and SS) with excellent intraclass correlation of > 0.950. Mean values from two researchers were then used for further analysis. Results for PD-1 had more variation between researchers and were separately re-reviewed. Cut-off values for high CD3 and CD8 lymphocyte counts were obtained from ROC (receiver operating characteristic) curves drawn in relation to 5-year overall survival. Cut-off values were 235 cells/mm² for CD3, 139 cells/mm² for CD8, and 19 cells/mm² for PD-1 from the tumor center and 744 cells/mm² for CD3 and 272 cells/mm² for CD8 from the invasive margin. The ROC curve for PD-1 from the invasive margin was left insignificant, and a specific cut-off value could not be determined. The prognostic effect was evaluated using cell number quartiles. As presented earlier in colon cancer with TMA samples (15), tumors were classified to form an Immune Cell Score (ICS) based on CD3 and CD8 lymphocyte densities. The ICS is based on the original Immunoscore (16), where high densities of CD3+ or CD8+ lymphocytes from samples of either the tumor center or invasive border are valued as one point. If both CD3+ and CD8+ lymphocytes have high densities in the tumor center and invasive margin, the ICS is 4, and if all samples have low cell densities, the ICS is 0. The ICS was then categorized into low (ICS 0-2) and high (ICS 3-4) groups. PD-L1 expression was evaluated on tumor cells (TC) and tumor-infiltrating immune cells (IC) by visually examining all individual tumor center samples, as presented by Ahtiainen et al. (13) The expression percentage used was the mean value of the samples and expression rate of at least 1% was considered positive. ICS and PD-L1^IC^ were further classified into four subtypes describing cancer microenvironment as proposed earlier (13, 17). ICS^high^/PD-L1^IChigh^ tumors have adaptive immune resistance, ICS^high^/PD-L1^IClow^ have immune tolerance, ICS^low^/PD-L1^IClow^ are immunologically ignorant and ICS^low^/PD-L1^IChigh^ tumors have oncogenic pathway induction without significant antitumoral immune reaction. Similar classification was then formed according to ICS and PD-1+ immune cell densities from tumor center.

### Statistical analysis

Categorical data were compared using Pearson’s chi-square test. ROC-curves in relation to 5-year overall survival were used to obtain cut-off values for immune cell densities by applying the point nearest to the (0,1) corner of the ROC plane technique. The use of time-dependent ROC-curve to identify cut-off values for immune cell densities has been advised by a professional statistician (15). The Kaplan-Meier method was used to calculate overall survival (OS), and the log-rank test was used to compare differences. Kaplan-Meier analysis utilized the complete follow-up data, and the reported 5-year overall survival represents the survival rate at that specific timepoint. A P-value of <0.05 was considered statistically significant. Survival times were from the date of surgery to the time of death from any cause (event), or to the end of the follow-up. Multivariable Cox proportional hazards regression model was used to analyze prognostic factors for OS. Statistical analyses were performed using IBM SPSS Statistics (version 27.0; SPSS Inc., Chicago, IL, USA).

## Results

Forty-seven patients were included in the analysis. Median age of patients was 65 years (interquartile range, IQR 53-69) with a slight predominance of the male gender (55%). The median overall survival in Kaplan-Meier analysis was 2.97 years (95% confidence interval, CI, 1.28-4.65). In this patient series, there were no post-operative deaths. Only four patients had other than cancer-related death during follow-up. Five patients (11%) had postoperative Clavien-Dindo (CD) grade 3b (requiring intervention under general anesthesia) or 4a (single organ dysfunction) complication. Three patients (6%) had CD3a (intervention without general anesthesia) and 39 (83%) had mild CD1-2 or no complications. Three patients (6%) had preoperative neoadjuvant therapy, and nine (20%) received adjuvant chemotherapy. Tumor was intrahepatic in 18 (38%), perihilar in 22 (47%), and distal in seven (15%) patients. Radical surgery with clear margins (R0) was achieved in 34 (72%) tumors with 13 (28%) having R1 resection with < 1mm margin. None of the patients had liver cirrhosis or hepatitis C infection. One patient had hepatitis B. Only one tumor was identified as mismatch repair deficient through MLH1 immune staining. Four patients had type 2 diabetes. Only one patient had pre-existing choledochal cyst. None had history of autoimmune cholangitis and only one patient had experienced a pancreatitis.

Clinicopathological variables according to ICS are shown in **Table 1**. Differences for age, sex, T class, N class, TNM stage, tumor grade, tumor size, vascular or perineural invasion or neoadjuvant or adjuvant treatment were not observed between ICS^high^ and ICS^low^ tumors. In addition, the expression of PD-L1 on immune cells or tumor cells was not associated with ICS. Tumors with high ICS were more often located intrahepatically (73% of the ICS^high^ tumors vs. 22% of ICS^low^) while ICS^low^ tumors were mostly perihilar (66%), P<0.001. R0 resection was achieved more often in ICS^high^ tumors [R1 resection in 12 (38%) of the ICS^low^ and only in one (7%) ICS^high^ tumor, P=0.028]. Only two (13%) of the ICS^high^ vs. 16 ICS^low^ (50%) needed preoperative stenting, and consequently, the preoperative bilirubin level was higher in ICS^low^ tumors [>16 in two (14%) of the ICS^high^ and 19 (63%) of the ICS^low^ tumors, P=0.002]. High PD-1 expression was significantly more common in ICS^high^ tumors (n=7, 47%) compared to ICS^low^ tumors (n=5, 16%), P=0.023.

**Table 1:** Clinicopathological variables according to immune cell score.

	ICS low total N=32 (% of column)	ICS high total N=15 (% of column)	Total N=47 (% of column)	P
Age				
<65 ≥65	18 (56) 14 (44)	7 (47) 8 (53)	25 (53) 22 (47)	0.539
Sex				
Male Female	20 (63) 12 (38)	6 (40) 9 (60)	26 (55) 21 (45)	0.148
T				
1 2 3	4 (13) 17 (57) 9 (30)	2 (13) 12 (80) 1 (7)	6 (13) 29 (64) 11 (22)	0.193
N				
0 1	24 (75) 8 (25)	10 (67) 5 (33)	34 (72) 13 (28)	0.552
Stage				
I II III	3 (10) 17 (55) 11 (35)	2 (13) 9 (60) 4 (27)	5 (11) 26 (57) 15 (33)	0.813
Tumor grade				
1 2 3	16 (52) 9 (29) 6 (19)	11 (79) 3 (21) 0 (0)	27 (60) 12 (27) 6 (13)	0.130
Tumor size				
< 5 cm ≥ 5 cm	19 (68) 9 (32)	7 (50) 7 (50)	26 (62) 16 (38)	0.261
Vascular invasion				
no yes	14 (44) 18 (56)	6 (40) 9 (60)	20 (43) 27 (57)	0.808
Perineural invasion				
no yes	3 (9) 29 (91)	3 (20) 12 (80)	6 (13) 41 (87)	0.309
Location				
intrahepatic perihilar distal	7 (22) 21 (66) 4 (13)	11 (73) 1 (7) 3 (20)	18 (38) 22 (47) 7 (15)	<0.001
Radicality of surgery				
R0 R1	20 (63) 12 (38)	14 (93) 1 (7)	34 (72) 13 (28)	0.028
PD-1				
low high	27 (84) 5 (16)	8 (53) 7 (47)	35 (74) 12 (26)	0.023
PD-L1 expression on immune cells				
< 1% ≥ 1%	23 (72) 9 (28)	10 (67) 5 (33)	33 (70) 14 (30)	0.716
PD-L1 expression on tumor cells				
< 1% ≥ 1%	30 (94) 2 (6)	13 (87) 2 (13)	43 (91) 4 (9	0.417
Preoperative stenting				
no yes	16 (50) 16 (50)	13 (87) 2 (13)	29 (62) 18 (38)	0.016
Preoperative bilirubin				
≤ 16 > 16	11 (37) 19 (63)	12 (86) 2 (14)	23 (52) 21 (48)	0.002
Neoadjuvant treatment				
No Yes	31 (97) 1 (3)	13 (87) 2 (13)	44 (94) 3 (6)	0.182
Adjuvant treatment				
No Yes	26 (87) 4 (13)	10 (67) 5 (33)	36 (80) 9 (20)	0.114

PD-1, programmed cell death protein 1; PD-L1, programmed cell death ligand-1.

Tumor stage was unknown in one, tumor grade was unknown in two, tumor size was unknown in five, and preoperative bilirubin level was unknown in three tumors.

### Univariable survival analysis

**Figure 2:**
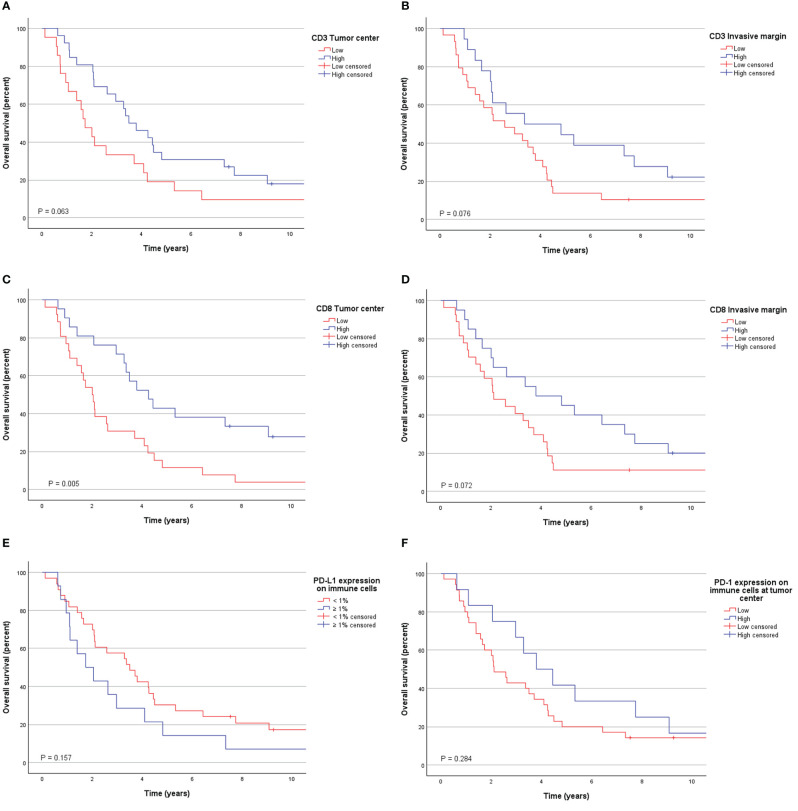
Overall survival according to **(A)** CD3 lymphocyte densities at the tumor center, **(B)** CD3 lymphocyte densities at the invasive margin, **(C)** CD8 lymphocyte densities at the tumor center, **(D)** CD8 lymphocyte densities at the invasive margin, **(E)** PD-L1 expression on immune cells at the tumor center, and **(F)** PD-1 expression on immune cells at the tumor center.

Survival according to different clinicopathological variables is shown in **Table 2**. None of the traditional prognostic factors, such as stage, tumor grade, vascular or perineural invasion, or radicality of surgery, had a significant effect on survival according to the Kaplan-Meier analysis. Preoperative stenting had a trend for worse survival (5-year OS 6% with stent and 38% without, P=0.068). Bilirubin level > 16 mg/dL was prognostic for worse survival (5-year OS 10% for bilirubin >16 mg/dL and 44% for bilirubin ≤ 16 mg/dL, P=0.037), but the bilirubin level was unknown in three patients. **Figure 2**. shows Kaplan-Meier graphs according to different immune factors.

**Table 2:** Overall survival according to clinicopathological variables.

	5-year overall survival		P
	N	%	
Age			
<65 ≥65	25 22	24% 27%	0.820
Sex			
Male Female	26 21	19% 33%	0.246
T			
1 2 3	6 29 10	33% 28% 20%	0.715
N			
0 1	34 13	24% 31%	0.788
Stage			
I II III	5 26 15	20% 19% 40%	0.618
Tumor grade			
1 2 3	27 12 6	26% 33% 17%	0.904
Tumor size			
< 5 cm ≥ 5 cm	26 16	35% 19%	0.130
Vascular invasion			
no yes	20 27	30% 22%	0.887
Perineural invasion			
no yes	6 41	33% 24%	0.506
Location			
intrahepatic perihilar distal	18 22 7	39% 23% 0%	0.502
Radicality of surgery			
R0 R1	34 13	27% 23%	0.648
PD-L1 expression on immune cells			
< 1% ≥ 1%	33 14	30% 14%	0.157
PD-1 expression on immune cells			
low high	35 12	20% 42%	0.284
CD3 from tumor center			
low high	21 26	19% 31%	0.063
CD8 from tumor center			
low high	26 21	12% 43%	0.005
CD3 from invasive margin			
low high	29 18	14% 44%	0.076
CD8 from invasive margin			
low high	27 20	11% 45%	0.072
Immune cell score			
low high	32 15	13% 53%	0.011
ICS/PD-L1IC groups			
ICS^high^/PD-L1^IChigh^ ICS^high^/PD-L1^IClow^ ICS^low^/PD-L1^IClow^ ICS^low^/PD-L1^high^	5 10 23 9	40% 60% 17% 0%	0.004
ICS/PD-1 groups			
ICS^high^/PD-1^high^ ICS^high^/PD-1^low^ ICS^low^/PD-1^low^ ISC^low^/PD-1^high^	7 8 27 5	57% 50% 11% 20%	0.030
Preoperative stenting			
no yes	29 18	38% 6%	0.068
Preoperative bilirubin			
≤ 16 > 16	23 21	44% 10%	0.037
Adjuvant treatment			
No Yes	36 9	22% 44%	0.277

**Figure 3:**
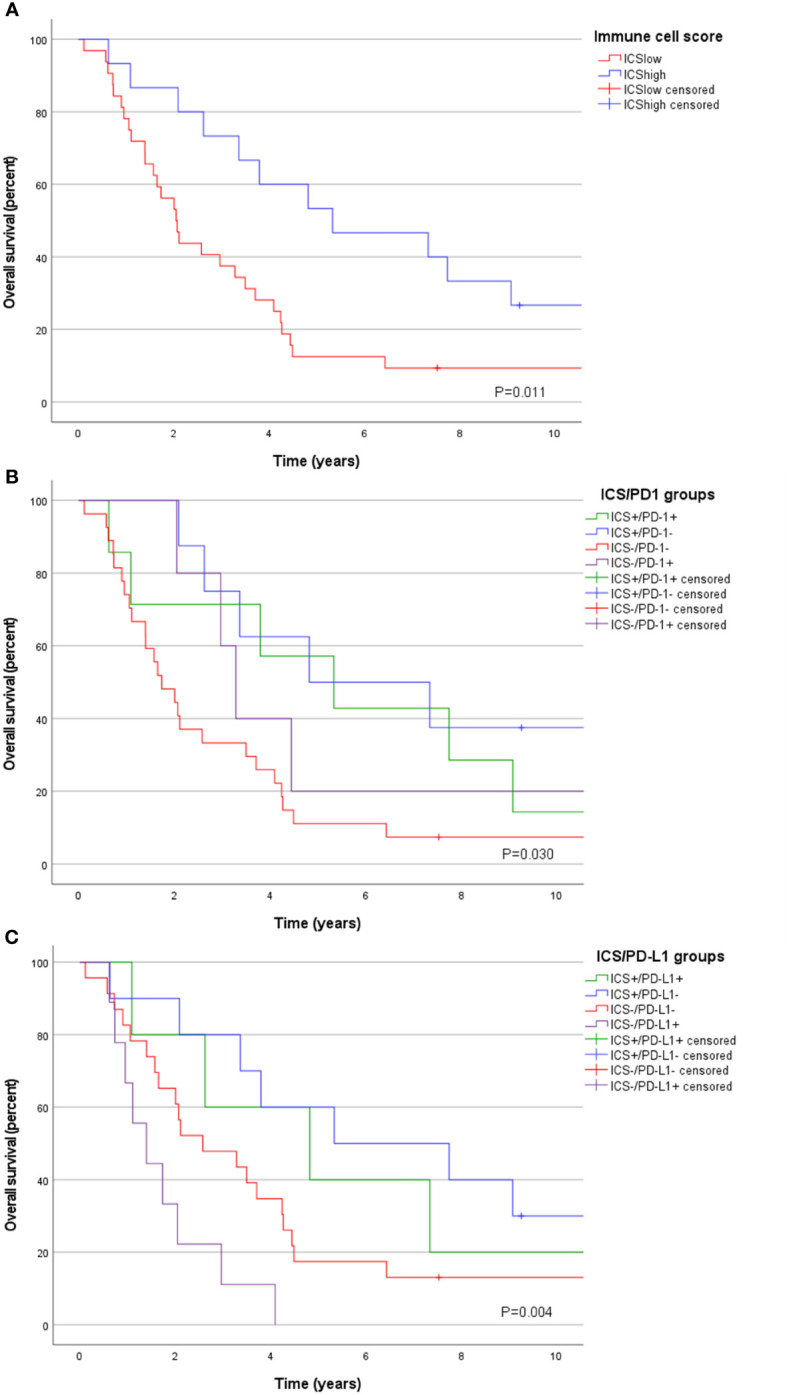
Overall survival according to **(A)** Immune cell score (ICS), **(B)** ICS/PD-1 groups, and **(C)** ICS/PD-L1^IC^ groups.

PD-1 expression on tumor invasive margin or on tumor center had no apparent significance on patient prognosis (P=0.942 and P=0.284, respectively). Low ICS was significantly associated with worse survival as 5-year OS was 13% for ICS^low^ and 53% for ICS^high^ tumors, P=0.011 (**Figure 3A**). When components of ICS were evaluated separately, the most effective prognostic factor was high CD8 lymphocyte infiltration on tumor center (5-year OS 43% for high density and 12% for low density tumors, P=0.005). Most of the tumors (n=27, 57%) were ICS^low^/PD-1^low^ and associated with a dismal prognosis as 5-year OS for those was only 11% compared to ICS^high^/PD-1^high^ and ICS^high^/PD1^low^ tumors with 5-year OS of 57% and 50% respectively, P=0.030 (**Figure 3B**). Similarly, most tumors were also ICS^low^/PD-L1^IClow^ (n=23, 49%) and associated with poor survival (**Figure 3C**). Positive PD-L1 expression on immune cells was associated with worse survival in ICS^low^ tumors (5-year OS for ICS^low^/PD-L1^IClow^ tumors was 17% vs. 0% for ICS^low^/PD-L1^IChigh^, P=0.026) but not in ICS^high^ tumor subgroup (P=0.563).

### Multivariable survival analysis

Age, sex, TNM stage, radicality of surgery, tumor location, and PD-L1^IC^ with ICS were selected for multivariable cox proportional hazards model shown in **Table 3**. Reference categories were age <65 years, female sex, TNM stage I, R0 resection, intrahepatic tumor location, and ICS^high^. ICS^low^ was a strong independent prognostic factor for worse survival with hazard ratio (HR) of 9.27 (95% CI 2.72-31.64), P<0.001. In addition, perihilar tumor location had some impact towards improved survival HR 0.21 (95% CI 0.06-0.73), p=0.048.

**Table 3:** Cox proportional hazards multivariable analysis.

	Univariable analysis		Multivariable analysis	
	HR (95% confidence interval)	P	HR (95% confidence interval)	P
Age				
<65 ≥65	1 0.93 (0.50-1.73)	0.820	1 1.00 (0.47-2.14)	0.991
Sex				
Male Female	1.45 (0.77-2.72) 1	0.249	2.04 (0.86-4.81) 1	0.105
TNM stage				
I II III	1 1.06 (0.36-3.12) 0.62 (0.19-2.06)	0.442	1 0.77 (0.22-2.71) 0.50 (0.13-1.90)	0.511
Radicality of surgery				
R0 R1	1 0.85 (0.41-1.73)	0.648	1 0.63 (0.27-1.48)	0.291
Tumor location				
intrahepatic perihilar distal	1 1.05 (0.52-2.09) 1.70 (0.66-4.36)	0.658	1 0.21 (0.06-0.73) 0.36 (0.09-1.35)	0.048
Immune cell score				
low high	2.46 (1.20-5.02) 1	0.014	9.27 (2.72-31.64) 1	< 0.001
PD-L1 expression at immune cells				
< 1% ≥ 1%	1 1.61 (0.83-3.14)	0.160	1 1.59 (0.68-3.70)	0.285

Three patients received neoadjuvant therapy and we performed additional analysis with this patient subgroup excluded. However, very little effect was seen on the results of univariable, or multivariable analysis and the ICS remained independent prognostic factor (HR for ICS^low^ 9.73, 95%CI 2.61-36.27, P<0.001, **Supplementary Table 1**).

## Discussion

Our results indicate that tumor infiltrating lymphocytes have significance in patient outcome also in cholangiocarcinoma as Immune cell score, based on CD3+ and CD8+ lymphocyte densities at tumor center and invasive margin, was a strong independent prognostic factor for overall survival when adjusted with age, sex, TNM stage, radicality of surgery, tumor location, and PD-L1^IC^ expression. According to univariable analysis CD8+ T-lymphocytes at tumor center had the most evident impact on patient outcome of the four ICS components. PD-1 or PD-L1 expression on immune cells did not have apparent impact on OS alone, but in ICS/PD-L1 subgroups analysis the PD-L1 positivity seemed to impair the survival for the ICS^low^ tumor patients.

Previous literature on prognostic impact of tumor infiltrating T-lymphocytes on CCA is contradictory (9). Wu ZY et al. presented a series of 76 patients with stage II and III tumors (21 intrahepatic and 55 extrahepatic) where CD3+ and CD8+ lymphocyte-based scoring was an independent prognostic factor surpassing TNM stage (18). Wu H et al. introduced a series of 50 patients with intrahepatic CCA, where Immunoscore-based scoring had only marginal prognostic value (P=0.048) in a multivariable model (19). A study by Goeppert et al. presented tumor grade and stage independent prognostic effect of FOXP3+ regulatory T-cells and CD4+ lymphocytes in 157 extrahepatic and 69 gallbladder CCA. However, CD8+ lymphocytes did not present independent prognostic impact. Moreover, tumor-infiltrating lymphocytic infiltrate did not predict positive outcome in 149 intrahepatic CCA (20). In contradiction to that, Xu et al. found that CD8+lymphocytes from TMA samples of 140 intrahepatic CCA had independent positive prognostic value (21). Also, a smaller study of 69 intrahepatic CCA found that CD8+ lymphocytes at the outer border of the tumor had positive impact on OS (22). Another study of intrahepatic CCA with 53 patients found that CD3+ lymphocytes had a borderline significance (P=0.049) for independent positive impact on OS while FOXP3+ lymphocytes presented possible independent negative impact (P=0.044) (23). For extrahepatic CCA, Kitano et al. presented in 114 tumors that CD8+ lymphocytes had prognostic value in univariable analysis and independently as a part of immune signature consisting of several immune cell populations (24). Oshikiri et al. studied 58 extrahepatic CCAs and found that high infiltration of CD8+ lymphocytes were prognostic for improved survival also in multivariable analysis (25). Ueno et al. showed in 117 extrahepatic CCA, that while high amount of CD8+ lymphocytes were associated with node-negative cancer, independent prognostic impact of only high infiltration of CD4+lymphocytes was observed (26).

PD-1/PD-L1 pathway is recognized as one of the most important tumor escape mechanisms and the field of cancer immunotherapy is rapidly growing. Established biomarkers predicting efficacy of PD-1 blockade therapy include expression of PD-L1 on tumors, high tumor mutation burden and microsatellite instability. Also, the abundance of CD8+ and B lymphocytes and expression of PD-L1 on tumor-infiltrating immune cells are associated with response to PD-1 blockade (10). Some evidence suggests that even very low (1%) PD-L1 positivity may be sufficient to predict the treatment efficiency (27). Normally PD-1 acts as an important inhibitor of both adaptive and innate immune responses to promote self-tolerance and is expressed mostly on activated T- and B-lymphocytes, but also on natural killer cells, macrophages, and dendritic cells (28). Binding of PD-L1 with PD-1 expressed by T-cells results in T cell anergy, exhaustion, apoptosis, or differentiation into regulatory function (29). In cancer microenvironment PD-L1 is expressed by not only tumor cells hiding immune surveillance but also by immune cells consisting mostly of peritumoral macrophages (30). PD-L1 expression on CCA tumor cells seems uncommon and is observed usually in tumors with high intratumoral lymphocytic infiltration (26, 31–33). In concordance to this, we found only four (9%) tumors with PD-L1^TC^ expression of at least 1% and only 1 (2%) expressed PD-L1^TC^ at least 5%. PD-L1^IC^ was somewhat more common with 1% expression rate seen in 14 (30%) and 5% expression rate in six (12%) tumors. However, in our study most CCAs were immunologically inactive tumors with ICS^low^/PD-1^low^ and ICS^low^/PD-L1^IClow^ subtype while only five tumors had ICS^high^/PD-L1^IChigh^ and might theoretically have responded to PD-1 blockade therapy (17).

Cancer microsatellite instability is usually associated with strong antitumoral immune response and more favorable prognosis but in CCA it is infrequently seen (33). In this study, we screened tumors for possible MMR deficiency by using MLH1 staining which should identify most of the tumors because of the strongest association of biliary tract cancers to *MLH1* Lynch syndrome (34, 35). Only one MMR deficient tumor was found, and the tumor microenvironment had ICS^low^/PD-L1^IChigh^ subtype associated with worst survival.

This study has some limitations. CCA is rare cancer and consequently the study population was relatively small. Also, immunohistochemical analysis were performed from TMA samples allowing more limited comprehension of tumor microenvironment compared to whole slide samples. Nevertheless, TMA-based immune cell analysis has proved useful and reliable for survival analysis (15, 16, 36). The patients were operated during 1990-2013, and evolution of treatment strategies cause a potential confounding time trend. Additionally, the use of adjuvant chemotherapy was seen here only in 20% of the patients as the benefit from adjuvant therapy in resected biliary tract cancer was not demonstrated until the BILCAP trial in 2019 (37). However, CCA is an extremely aggressive cancer with startling mortality, and we provide additional information on the limited understanding of the CCA immune environment.

Evaluation of the CCA immune contexture provides useful prognostic information as ICS was a strong independent prognostic factor. Selected patients with CCA might benefit from immune quantification to guide immunotherapy with PD-1/PD-L1 pathway blockade therapy.

## Data availability statement

The datasets presented in this article are not readily available because of Finnish laws of privacy protection. The data are available upon request and acquisition of ethical and institutional approval for data transfer. Requests to access the datasets should be directed to https://finbb.fi/en/fingenious-service.

## Ethics statement

The studies involving humans were approved by the Helsinki University Hospital’s ethical committee, Helsinki University Hospital institutional review board, and Helsinki Biobank. The studies were conducted in accordance with the local legislation and institutional requirements. The ethics committee/institutional review board waived the requirement of written informed consent for participation from the participants or the participants’ legal guardians/next of kin because of the institutional approval for a biobank study.

## Author contributions

E-VW: Conceptualization, Data curation, Formal analysis, Investigation, Methodology, Validation, Visualization, Writing – original draft, Writing – review & editing. SS: Formal analysis, Investigation, Validation, Writing – review & editing. HK: Data curation, Investigation, Validation, Writing – review & editing. AN: Resources, Writing – review & editing. HM: Resources, Writing – review & editing. JA: Data curation, Resources, Writing – review & editing. JS: Resources, Writing – review & editing. MA: Data curation, Investigation, Methodology, Validation, Writing – review & editing. JB: Project administration, Resources, Supervision, Writing – review & editing. J-PM: Conceptualization, Funding acquisition, Project administration, Supervision, Writing – review & editing. VS: Conceptualization, Data curation, Funding acquisition, Project administration, Supervision, Writing – review & editing. TS: Conceptualization, Data curation, Funding acquisition, Methodology, Project administration, Supervision, Writing – original draft, Writing – review & editing.
