# Supplementary material for: High immune cell infiltration predicts improved survival in cholangiocarcinoma
Source: Front Oncol. 2024 May 1;14:1333926. doi: 10.3389/fonc.2024.1333926 (PMC11094285; doi:10.3389/fonc.2024.1333926)
Supplement: Supplementary file 1 [file Table_1.docx]

|  | Univariable analysis |  | Multivariable analysis | |
| --- | --- | --- | --- | --- |
|  | HR (95% confidence interval) | P | HR (95% confidence interval) | P |
| **Age:**  <65  ≥65 | 1  0.95 (0.50-1.82) | 0.880 | 1  1.07 (0.47-2.45) | 0.868 |
| **Sex:**  Male  Female | 1.64 (0.85-3.18)  1 | 0.144 | 2.04 (0.86-4.88)  1 | 0.107 |
| **TNM stage:**  I  II  III | 1  1.24 (0.42-3.62)  0.94 (0.30-2.97) | 0.747 | 1  0.71 (0.20-2.59)  0.49 (0.13-1.87) | 0.521 |
| **Radicality of surgery:**  R0  R1 | 1  0.85 (0.41-1.76) | 0.662 | 1  0.63 (0.27-1.48) | 0.288 |
| **Tumour location:**  intrahepatic  perihilar  distal | 1  1.09 (0.52-2.29)  1.79 (0.67-4.80) | 0.472 | 1  0.20 (0.05-0.74)  0.37 (0.09-1.46) | 0.050 |
| **Immune cell score:**  low  high | 2.51 (1.16-5.42)  1 | 0.019 | 9.73 (2.61-36.27)  1 | < 0.001 |
| **PD-L1 expression at immune cells:**  < 1 %  ≥ 1% | 1  1.46 (0.72-2.97) | 0.293 | 1  1.48 (0.61-3.59) | 0.391 |
